# A High Resolution DNA Melting Curve Analysis for the Rapid and Efficient Molecular Diagnostics of Extended Spectrum β-Lactamase Determinants from Foodborne *Escherichia coli*

**DOI:** 10.3390/microorganisms8010090

**Published:** 2020-01-09

**Authors:** Patrick Murigu Kamau Njage, Elna Buys

**Affiliations:** 1 Division for Epidemiology and Microbial Genomics, National Food Institute, Technical University of Denmark, Kemitorvet, Building 204, 2800 Kongens Lyngby, Denmark; 2 Department of Consumer and Food Sciences, University of Pretoria, Lynwood Road, Pretoria 0002, South Africa

**Keywords:** *Escherichia coli*, antibiotic resistance, extended-spectrum β-Lactamases, genotyping, high-resolution DNA melting curve, real-time PCR

## Abstract

The accurate identification of Extended-Spectrum β-Lactamase (ESBL) genes in Gram-negative bacteria is necessary for surveillance and epidemiological studies of transmission through foods. We report a novel rapid, cheap, and accurate closed tube molecular diagnostic tool based on two multiplex HRM protocols for analysis of the predominant ESBL families encountered in foods. The first multiplex PCR assay targeted blaCTX-M including phylogenetic groups 1 (CTX-M-1-15, including CTX-M-1, CTX-M-3 and CTX-M-15), 2 (CTX-M-2), and 9 (CTX-M-9-14, including CTX-M-9 and CTX-M-14). The second assay involved blaTEM /bla CTX-M /blaSHV, including TEM variants (TEM-1 and TEM-2), SHV-1-56 (SHV-1, SHV-2 and SHV-56), and CTX-M-8-41 (CTX-M-8, CTX-M-25, CTX-M-26 and CTX-M-39 to CTX-M-41). The individual melting curves were differentiated by a temperature shift according to the type of ESBL gene. The specificity and sensitivity of the first assay were 100% and 98%, respectively. For the second assay, the specificity and sensitivity were 87% and 89%, respectively. The detection of ESBL variants or mutations in existing genes was also demonstrated by the subtyping of a variant of the CTXM-1-15. The HRM is a potential tool for the rapid detection of present β-lactamase genes and their characterization in a highly sensitive, closed-tube, inexpensive method that is applicable in high throughput studies.

## 1. Introduction

Antibiotics belonging to the class β-lactams have been instrumental in both human and veterinary medicine for the treatment of infections by Gram-negative pathogens [1]. Elaboration of β-lactamases has adversely affected the treatment of infections caused by Gram-negative pathogens due to the ability of these enzymes to inactivate β-lactam antibiotics by hydrolysis [2,3]. Extended-spectrum or third-generation cephalosporins have been in the past, effective against β-lactam resistant pathogens. However, several Gram-negative pathogens developed the ability to produce mutant forms of the “older” β-lactamases, also called extended-spectrum β-lactamases (ESBLs). These mutant forms of β-lactamases are able to hydrolyze the new-generation cephalosporins and aztreonam [4].

ESBLs can be classified as class A β-lactamases mainly from the three main families, TEM, SHV, and CTX-M [5]. Another closely related but distinct group of β-lactamases includes the AmpC β-lactamases, which confer resistance to additional β-lactams and β-lactamase inhibitors [6]. β-lactams that are commonly hydrolyzed by ESBL and AmpC β-lactamases belong to a group of critically important antimicrobials in both human and animal medicine [7]. This has resulted in increased morbidity, mortality, and health care costs all over the world [8].

Increasing evidence implicate animal-based foods and also recently plant-based foods as a source of ESBL resistant bacteria spread through consumption or cross-contamination, as well as the transfer of resistance genes [9,10,11,12,13]. We recently reported the role of the production environment in exposure of the consumer with considerably high ESBL positive *E. coli* levels in vegetables from both initially ESBL positive *E. coli* as well as newly ESBL positive *E. coli* due to gene transfer [14,15].

Various methods have been used for the isolation, identification, and characterization of ESBL and or AmpC producing bacteria. Selective isolation of ESBL and/or AmpC-producers from feces or foods has been performed through the addition of 3rd generation cephalosporins to selective or non-selective growth media. Selective isolation in foods is usually followed by confirmation of the ESBL and AmpC genes by either phenotypic or molecular methods. Phenotypic tests have been based on disc diffusion or dilution assays. The synergy between combinations of 3rd generation cephalosporin and clavulanic acid indicates the presence of an ESBL gene. A disadvantage of this approach is that resistant strains may contain several ESBL and AmpC genes, which could interfere with the result of the confirmatory phenotypic testing. Molecular or genotypic methods, therefore, present a more appropriate approach towards confirmation of the presence of an ESBL and/or AmpC producer. Such classical approaches tend to be labor-intensive, time-consuming, and less sensitive, precise, and reproducible. Dallenne et al. [16] described a polymerase chain reaction (PCR) based approach and primers targeting the most important β-lactamases in *Enterobacteriaceae*, including genes encoding the OXA-1-like broad-spectrum β-lactamases, ESBLs, plasmid-mediated AmpC β-lactamases, and class A, B, and D carbapenemases. Upon the identification of the ESBL and AmpC genes, subtyping can further be done by sequence analysis of PCR fragments.

Most of the methods in current use pose several challenges. Phenotypical tests based on disc diffusion or dilution assays involve manual steps and many hours of incubation. Most of the molecular genotyping and mutant subtyping approaches involve the use of gels or other matrices to separate and detect the mutants from the PCR products [17]. This introduces both the need for automation, further delays in obtaining results, and the enhanced possibility of laboratory contamination. High-resolution DNA melting analysis (HRM), which supports the simultaneous mutation scanning and genotyping of PCR products, is a potential mitigation approach to such disadvantages [17,18]. HRM works by the application of an asymmetric PCR conducted with the addition of a saturating fluorescent DNA dye and unlabeled oligonucleotide probes. This allows for the analysis of fluorescent melting curves of both PCR amplicons and amplicon-probe duplexes in a closed tube protocol [17,18].

Genotyping using HRM is also possible by the detection of changes in the shape and/ or melting temperature of the amplicon melting transition curve introduced by heterozygous sequence alterations [17]. Background subtraction and normalization are conducted before genotypes can be identified for known sequence variations by assessing probe-melting transitions. HRM approach enables the detection of even single base-pair change within the probe region because any such changes result in a significant shift in the melting temperature (Tm) of the probe-amplicon duplex, which supports the differentiation of both homozygous and heterozygous sequence variants [17]. Monitoring fluorescence of saturating dye enables the identification of Tm where the Tm of DNA duplex is the temperature at which the normalized fluorescence is 50%. HRM presents a simple, low-cost, and rapid method to scan for known and unknown mutations [19], including a large number of samples. Recent real-time PCR instruments and intercalating dyes applied at saturating concentrations have enabled closed-tube discrimination of very subtle sequence changes in PCR amplicons [20].

HRM methods have been developed and applied for the identification and subtyping of bacterial and viral pathogens [19,21], genes encoding selected β-lactamases from hospital settings [22,23], AmpC β-lactamases [24], Metallo-β-Lactamase [25], and multidrug-resistant *Mycobacterium tuberculosis* [20] mostly of clinical microbiology concern. The most prevalent ESBLs and their variants associated with foods and food animals [15] differ from those targeted by HRM protocols developed for isolates from clinical settings. It is, therefore, important to explore the potential of HRM for the detection of ESBL genes commonly associated with food isolates.

This study aimed to develop a rapid and accurate multiplex HRM molecular diagnostic tool for typing of frequently detected Extended Spectrum β-lactamase determinants from *E. coli* isolates of food origin.

## 2. Materials and Methods

### 2.1. E. coli Isolates

A well-characterized panel of β-lactamase-producing strains was used as controls for the development and optimization of the HRM assays. These isolates included 46 non-repetitive *Escherichia coli* strains, which were previously isolated from lettuce and irrigation water [26] and typed phenotypically for ESBL/AmpC, followed by confirmation of specific genes using multiplex PCR and amplicon sequencing [15]. *Escherichia coli* ATCC 25922 (ESBL negative), *E. coli* ATCC 35218 (ESBL positive control), *K. pneumoniae* ATCC 700603 (ESBL positive), and *Pseudomonas aeruginosa* ATCC 27853 (ESBL negative) strains were used as control strains for test performance.

### 2.2. Design of Group-Specific Primers for HRM Assays

Two multiplex HRM PCRs were designed targeting the major ESBLs of concern in microbial food contaminants [15] and subdivision of ESBLs based on their amino acid identities [16]. The first assay involved a *bla*_CTX-M_ multiplex PCR targeting phylogenetic groups 1 (CTX-M-1-15 including CTX-M-1, CTX-M-3 and CTX-M-15), 2 (CTX-M-2), and 9 (CTX-M-9-14 including CTX-M-9 and CTX-M-14). The second assay involved *bla*_TEM_/*bla*_CTX-M_ /*bla*_SHV_, including TEM variants (TEM-1 and TEM-2), SHV-1-56 (SHV-1, SHV-2, and SHV-56) and CTX-M-8-41 (CTX-M-8, CTX-M-25, CTX-M-26 and CTX-M-39 to CTX-M-41). Sequences of these genotypes were downloaded from GenBank (National Center for Biotechnology Information, National Institutes of Health, Bethesda, MA, USA). Primers were designed after analysis and alignments of each ESBL type to amplify fragments below 300 bp (Table 1).

### 2.3. Multiplex PCR Technique

The glycerol stock *E. coli* isolates were streaked on blood agar plates to ensure colony purity followed by overnight incubation at 37 °C. A colony was suspended in 500 µL double distilled water. This solution was then heated at 95 °C for 10 min, followed by cooling and centrifugation of the cell suspension at 4 °C. The DNA containing supernatant was used for further assay development. Assay optimization was performed beginning with simplex ESBL group PCR followed by the multiplex format, which involved different primer pair concentrations. The MgCl_2_ concentration, which may greatly affect the annealing of heterozygotes and melting of the amplicons during HRM, was also optimized. The primer sequences, positions, and concentrations, and the sizes of the corresponding amplicons are given in Table 1.

Each of the 50 µL reactions contained 1× KAPA HRM PCR Master Mix (KAPA Biosystems, Wilmington, MA, USA), 3.5 mM MgCl_2_, 0.2 µL of each primer (Table 1) and 2 µL of DNA. The fluorescent saturating dye, EvaGreen (Kappa Biosystems, Wilmington, MA, USA) in the master mix supports the saturation of all double-stranded DNA molecules. PCR and HRM were performed using a CFX96 Touch™ Real-Time PCR Detection System (Biolabs New England, Ipswich, MA, USA).

Amplification was carried out as follows for the first multiplex HRM protocol: initial denaturation at 95 °C for 3 min; 30 cycles of 95 °C for 5 s, 57 °C for 30 s, and 95 °C for 1 min; and a final elongation step at 72 °C for 7 min. The optimal annealing temperature was at 58 °C for the second HRM multiplex protocol. A melt curve step after the initial amplification included gradually increasing the temperature from 65 °C to 90 °C at 0.5 °C/s with data acquisition after every second.

The melting curves were analyzed by background removal and normalization. The melting temperature Tm was calculated as the temperature at which the normalized fluorescence is 50%. Melt curves were converted into melting peaks by plotting the negative derivative of relative fluorescence (RFU) versus temperature (-d(RFU)/dT), which can be visualized as peaks representing the Tm of the double-stranded DNA complexes. Experiments were repeated three times in duplicates.

### 2.4. Assay Accuracy, Validation, and Sensitivity

Accuracy of amplification was checked by agarose gel electrophoresis. For visualization, amplicons were run on 1.6% agarose gel containing 10,000× SYBR Safe DNA stain concentrate (Invitrogen, Carlsbad, CA, USA) diluted 1:10,000 in agarose gel buffer. For confirmation of positive ESBL/AmpC β-lactamase gene groups from the assay, bidirectional sequencing of PCR products from selected isolates was performed after the excision of bands from the gel followed by DNA cleaning and concentration (Zymo Research, Irvine, CA, USA). The gene sequences were explored with the software FinchTV version 1.4.0 (Geospiza, Seattle, WA, USA) and aligned using BioEdit [27]. A comparison with the available databases was made using the National Center for Biotechnology Information database matching (Available online: http://blast.ncbi.nlm.nih.gov/Blast.cgi). Phylogenetic trees were plotted for the sequenced amplicons together with sequences of reference genotypes downloaded from GenBank. The evolutionary history was inferred using the neighbor-Joining method [28]. The optimal tree with the sum of branch length = 2.023 is presented in the results section.

The percentage of replicate trees in which the associated taxa clustered together in the bootstrap test (1000 replicates) was shown next to the branches [29]. The trees were plotted to scale, with branch lengths in the same units as those of the evolutionary distances used to infer the phylogenetic tree. The evolutionary distances were computed using the Jukes–Cantor method [30] in the units of the number of base substitutions per site. Codon positions included were 1st + 2nd + 3rd + Noncoding. All ambiguous positions were removed for each sequence pair. Phylogenetic analyses were conducted in MEGA5 [31]. The accuracy of the assay was assessed by running the HRM assay on all the 46 strains and comparing it with data obtained from Njage and Buys [15] to calculate the sensitivity and specificity of the HRM assays.

## 3. Results and Discussion

ESBL detection in foodborne Gram-negative bacteria relies on phenotypic tests based on disc diffusion or dilution tests, which involves a significant delay in results due to manual steps and incubation time. Further application of molecular genotyping and subtyping approaches also involve substantial manual handling with introduces further delays and increased possibility of contamination. We have developed an HRM protocol for rapid, closed tube detection of ESBL through two multiplex PCR protocols. These protocols are seamlessly followed by melting curve analysis relying on a saturating fluorescent DNA dye for the detection of common ESBLs from food isolates [15] targeting 17 ESBL genes from CTX-M, TEM, and SHV groups.

**Figure 1:**
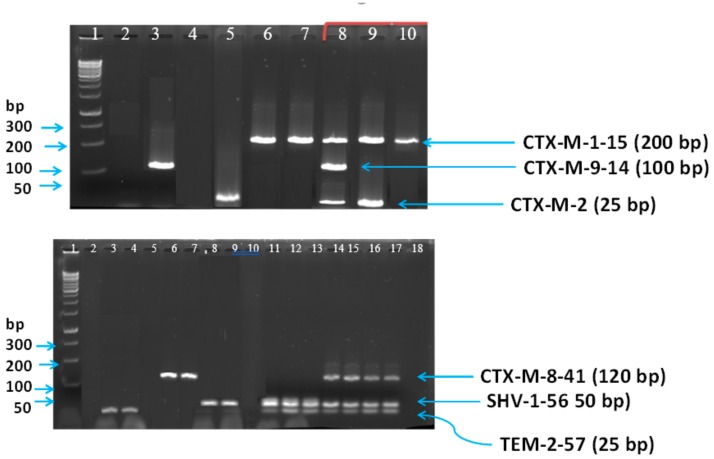
Illustrative multiplex PCR *bla*_CTX-M_ (CTX-M-1-15 including CTX-M-1, CTX-M-3 and CTX-M-15; CTX-M-2 including CTX-M-2; CTX-M-9-14 including CTX-M-9 and CTX-M-14), *bla*_TEM_ */bla*_TEM_*/bla*_SHV_ including TEM variants (TEM-1 and TEM-2), SHV-1-56 (SHV-1, SHV-2 and SHV-56) and CTX-M-8-41 (CTX-M-8, CTX-M-25, CTX-M-26 and CTX-M-39 to CTX-M-41). Top panel: Lanes 1, DNA ladder; 2, RNAse free sterile water; 3, isolate with *CTX-M-9-14*; 4, ESBL negative *Escherichia coli* ATCC 25922; 5, isolate with *CTX-M-2*; 6–7, isolates with *CTX-M-1-15*; 8, isolate with *CTX-M-1-15, CTX-M-9-14*, and *CTX-M-2*; 9, isolate with *CTX-M-1-15 and CTX-M-2*; 10, isolate with *CTX-M-1-15*. Bottom panel Lanes 1, DNA ladder; 2, RNAse free sterile water; 3–4, isolates with *TEM-2-57*; 5, ESBL negative *Escherichia coli* ATCC 25922; 6–7, isolate with *CTX-M-8-41*; 8–9, isolates with *SHV-1-56*; 10, isolate with none of the three genes; 11–12, isolates with *SHV-1-56 and TEM-2-57*; 13–17, isolates with *CTX-M-8-41, SHV-1-56,* and *TEM-2-57*; 18, ESBL negative *Pseudomonas aeruginosa* ATCC 27853.

The initial part of the study involved the optimization of single and multiplex PCR protocols for the detection of ESBL genes. The positive and negative strains yielded positive or negative amplicons, respectively, as illustrated in the gel electrophoresis for both single and combined ESBL from the initial optimization of the multiplex PCR technique (Figure 1).

For the first assay, the gene targets (amplicon sizes) were CTX-M-1-15 (200 bp), CTX-M-9-14 (100 bp), and CTX-M-2 (25 bp). The second assay consisted of the gene targets (molecular weights) CTX-M-8-41 (120 bp), SHV-1-56 (50 bp), and TEM-2-57 (25 bp). Isolates containing more than one gene were also distinguishable by the multiplex PCR.

**Figure 2:**
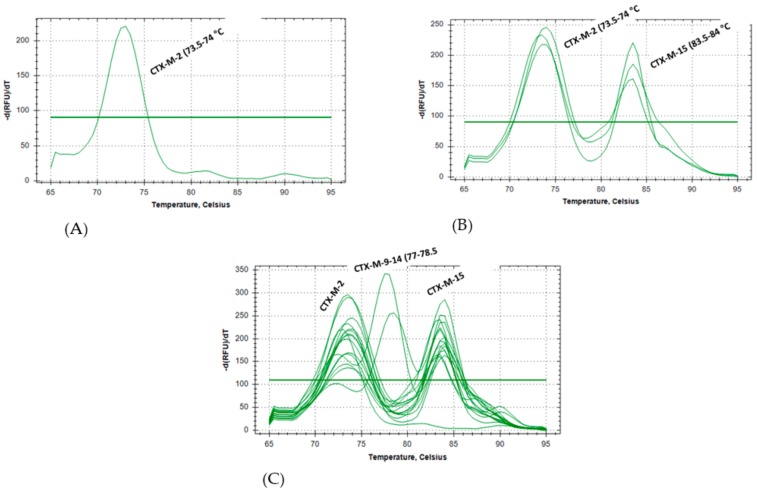
Illustrative plot showing detection of CTX-M through HRM analysis of *E. coli* isolates (**A**) an isolate with CTX-M-2, (**B**) three isolates with both CTX-M-2 and CTX-M-15, (**C**) 14 isolates with both CTX-M-2 + CTX-M-15, and two isolates with CTX-M-9. Melting temperatures (Tm) are shown for each HRM target.

**Figure 3:**
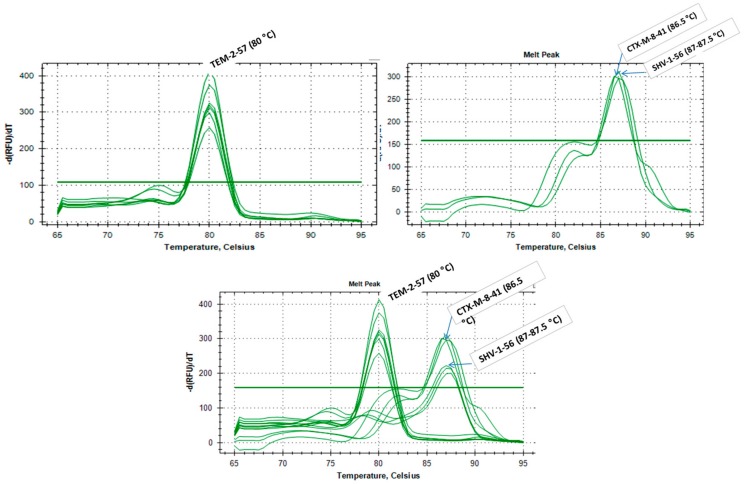
Illustrative plot showing detection of CTX-M, SHV and TEM through HRM analysis of *E. coli* isolates (**A**) simplex HRM for seven isolates with TEM-2-57, (**B**) duplex HRM for two isolates with SHV-1-56 and one with CTX-M-8-41, (**C**) triplex HRM for seven isolates with TEM-2-57, five isolates with SHV-1-56 and one isolate with CTX-M-8-41. Melting temperatures (Tm) are shown for each HRM target.

Subsequent steps involved the development of HRM protocols for the identification and subtyping of the ESBL genes and their variants. Illustrative plots of the normalized melt curves for the first and the second multiplex assays are shown in Figure 2 and Figure 3, respectively. The melt curves successfully detected and differentiated the genes from isolates containing the single and multiple genes. For the first multiplex assay, ESBL genes CTX-M-2, CTX-M-15, and CTX-M-9-14 showed distinct melting temperatures at 73.5–74 °C, 83.5–84 °C, and 77–78.5 °C respectively (Figure 2).

The second assay showed the TEM-2-57, CTX-M-8-41, and SHV-1-56 at melting temperatures of 80 °C, 86.5–87 °C, and 87–87.5 °C respectively (Figure 3). There was, therefore, an overlap in the CTX-M-8-41 upper melting temperature with the lower range for SHV-1-56 both at 87 °C. These gene targets can be detected in below 3 h after DNA extraction without any further manual steps after set-up. The universal nature of group-specific ESBL primers allows for further differentiation of ESBL genes belonging to these groups without the use of further typing methods such as restriction analysis or direct sequencing. For instance, the group CTX-M-1-15 contains the gene targets CTX-M-1, CTX-M-3 and CTX-M-15 which could all be detected using the group-specific assay. This was also possible for other ESBL groups. HRM assays have also been previously developed for the typing and subtyping of both microbial species and β-lactamases, most frequently isolated from certain hospital settings. For instance, HRM protocols were developed and tested for detection of TEM, SHV, and CTX-M beta-lactamases in *E. coli*, *Klebsiella pneumoniae*, and *Enterobacter cloacae* from patients admitted in hospitals [21,22] and for detection and identification of metallo-β-lactamases [24]. Chia et al. [22] developed an efficient multiplex PCR targeting bla(SHV), bla(CTX-M-3)-like, and bla(CTX-M-14)-like genes and a modified SHV melting-curve mutation detection method to rapidly distinguish six prevalent bla(SHV) genes (bla(SHV-1), bla(SHV-2), bla(SHV-2a), bla(SHV-5), bla(SHV-11), and bla(SHV-12)) in Taiwan hospitals. Chromá et al. [23] developed a multiplex polymerase chain reaction and melting curve analysis for the detection and discrimination of β-lactamases commonly detected in isolates from intensive care patients. HRM approaches have also been developed for the detection of non-ESBL β-lactamase genes such as plasmid-mediated AmpC β-lactamase genes [24] and metallo-β-lactamase-encoding genes [25] from clinical strains. HRM protocols have also been used for rapid high-throughput detection and subtyping of Noroviruses [21], multidrug-resistant *Mycobacterium tuberculosis* [20], and frequently isolated Salmonella serovars [19]. Our results show that the HRM approach can also be used for the detection of common ESBLs in foodborne bacteria. 

**Figure 4:**
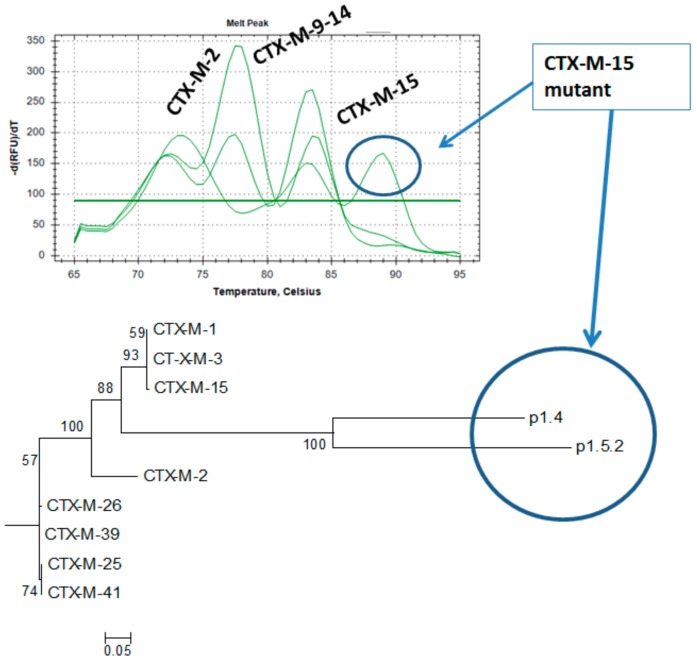
Detection of CTX-M-15 sequence mutant isolates p1.4 and p1.5.2 by postHRM sequencing of PCR amplicons for isolates, showing a new HRM peak at Tm of 89 °C.

Further interest would be the identification of new ESBL variants or mutations in existing genes. The utility of the HRM approach in the identification of new ESBL variants or mutations in existing genes can be demonstrated by the extra melting temperature and peak detected in some isolates at 89 °C. This was confirmed through sequencing and phylogeny tree containing variants of CTXM-1-15. Figure 4 shows the extra melting temperature and peak detected in some isolates at 89 °C. A phylogenetic tree is also shown depicting sequenced CTXM-1-15 amplicons from two *E. coli* isolates as well as GenBank references from the CTXM-1-15 and the CTX-M-41 groups. The amplicons from the *E. coli* isolates with Tm at 89 °C clustered at 100% with each other and at 88% with the CTXM-1-15 references from GenBank, indicating these were variants of CTXM-1-15 (Figure 4).

This HRM approach, therefore, further supported the detection of changes resulting in a significant shift in the melting temperature (Tm) of the probe-amplicon duplex. Further extension of the approach to other ESBL gene targets of relevance to foodborne bacteria is recommended for wider application of the method.

Accuracy of the assay was assessed against all the 46 strains and comparing with data obtained from Njage and Buys [15]. The specificity and sensitivity values were 100% and 98%, respectively, for the first HRM assay. For the 2nd assay, the specificity and sensitivity were 87% and 89%, respectively. Such a lower performance of the second assay may be attributed to the overlap in the CTX-M-8-41 upper melting temperature with the lower range for SHV-1-56 both at 87 °C

## 4. Conclusions

We have proposed a closed tube, rapid, accurate HRM method for typing of main Extended Spectrum β-lactamase determinants found in *E. coli* isolates from foods. This method presents the possibility for downstream sequence analysis and detection of new ESBL variants. The approach may be extended for ESBL gene targets of other Enterobacteriaceae prevalent in different food products.

Adoption of this technique in practice will require minimal additional investments in laboratories undertaking molecular typing of foodborne pathogens. The main requirements are real-time PCR instruments and intercalating dyes to enable the identification of Tm. These additional investments impose little extra investment in skills and cost. Potential for PCR inhibition and variation in relative amplification of PCR targets due to DNA quality are potential limitations that may be addressed by the incorporation of various common approaches to ensure good DNA quality.

## Figures and Tables

**Table 1:** Primers used in this study.

Protocol	Target Name	Gene Targets	Name	Sequence (5′–3′)	Expected Amplicon Length (bp)	GenBank Accession No.
Multiplex 1	CTX-M-1-15	CTX-M group 1: CTX-M-1, CTX-M-3 and CTX-M-15	CTX-M-1-15-F CTX-M-1-15-R	CGCAAATACTTTATCGTGCTGAT GATTCGGTTCGCTTTCACTTT	102	X92506 Y10278 AY044436
	CTX-M-2	CTX-M group 2: CTX-M-2	CTX-M-2-F CTX-M-2-R	CTGGTTCTGGTGACCTACTTTAC GCGATACCTCGCTCCATTTAT	124	X92507
	CTX-M-9-14	CTX-M group 9: CTX-M-9 and CTX-M-14	CTX-M-9-14-F CTX-M-9-14-R	GCTCATCGATACCGCAGATAAT CCGCCATAACTTTACTGGTACT	86	AF174129 AF252622
Multiplex 2	TEM-2-57	TEM variants: TEM-1 and TEM-2	TEM-2-57-F TEM-2-57-R	CGGATGGCATGACAGTAAGA TCCGATCGTTGTCAGAAGTAAG	89	X54606
	SHV-1-56	SHV-1, SHV-2 and SHV-56	SHV-1-56-F SHV-1-56-R	CTGGAGCGAAAGATCCACTATC CGCTGTTATCGCTCATGGTAA	130	AF148850 AF148851 EU586041
	CTX-M-8-41	CTX-M-8, CTX-M-25, CTX-M-26 and CTX-M-39 to CTX-M-41	CTX-M-8-41-F CTX-M-8-41-R	GAGCCGACGCTCAACAC CACYGCCCAACGTCAGATT	97	AF189721 AF518567 AY157676 AY954516 DQ023162
